# Cerebral toxoplasmosis and alcohol abuse in AIDS: dementia with multiple etiologies

**DOI:** 10.1590/1980-57642020dn14-040014

**Published:** 2020-12

**Authors:** Katie Moraes de Almondes, Nathalya Chrispim Lima

**Affiliations:** 1 Associate Professor at the Department of Psychology and on the Postgraduate Program in Psychobiology, Universidade Federal do Rio Grande do Norte - Natal, RN, Brazil.; 2 Postgraduate Program in Psychobiology, Universidade Federal do Rio Grande do Norte - Natal, RN, Brazil.

**Keywords:** neurocognitive disorders, AIDS dementia complex, toxoplasmosis cerebral, neuropsychology, cognition, behavior, transtornos neurocognitivos, complexo AIDS demência, toxoplasmose cerebral, neuropsicologia, cognição, comportamento

## Abstract

Major neurocognitive disorder due to multiple etiologies, or dementia due to multiple etiologies (DME), is a term coined by the Diagnostic and Statistical Manual of Mental Disorders to refer to complex cases when multiple pathologies, such as Alzheimer's disease, Lewy Bodies, human immunodeficiency virus (HIV), vascular-related brain damage or frontotemporal lobar degeneration, are identified as contributing to neurocognitive impairment and/or behavioral alterations, based on patient's neuroimaging tests, laboratorial exams, associated symptomatology and medical history. In this study, we report the case of a 63-year-old male patient who presented with parkinsonism symptoms, aphasia and cognitive impairment on multiple domains after cerebral toxoplasmosis related to acquired immunodeficiency syndrome, vascular damage and a history of alcohol abuse. We discuss the neurocognitive and neurobehavioral variables that characterized this diagnosis, as well as the importance of the differential diagnosis of DME on the field of neuropsychology of aging and, especially, for individuals living with HIV infection.

## RESUMO

Transtorno neurocognitivo maior devido a múltiplas etiologias, ou demência por múltiplas etiologias (DME), é um termo estabelecido pelo Manual Diagnóstico e Estatístico de Transtornos Mentais para se referir a casos complexos em que múltiplas patologias, como a Doença de Alzheimer, Corpos de Lewy, o vírus da imunodeficiência humana (HIV), danos de origem vascular ou a degeneração lobar frontotemporal, são identificados como contribuintes para o comprometimento neurocognitivo e/ou para alterações comportamentais, com base em testes de neuroimagem do paciente, exames laboratoriais, sintomatologia associada e histórico médico. Neste artigo, relatamos o caso de um paciente do sexo masculino de 63 anos que apresentou sintomas de parkinsonismo, afasia e comprometimento cognitivo em múltiplos domínios após neurotoxoplasmose relacionada à síndrome da imunodeficiência adquirida, dano vascular e histórico de abuso de álcool. Foram discutidas as variáveis neurocognitivas e neurocomportamentais que caracterizaram esse diagnóstico, assim como a importância do diagnóstico diferencial de DME para a neuropsicologia do envelhecimento e, especialmente, para indivíduos portadores do HIV.

## INTRODUCTION

Major neurocognitive disorder due to multiple etiologies, or dementia due to multiple etiologies (DME), is a term coined by the Diagnostic and Statistical Manual of Mental Disorders to refer to complex cases when multiple pathologies are identified as contributing to neurocognitive impairment and/or behavioral alterations, based on patient's neuroimaging tests, laboratorial exams, associated symptomatology and medical history (5^th^ ed.; DSM-5).^1^ Amongst the several possible etiologies for major neurocognitive impairment, human immunodeficiency virus (HIV) infection stands as one of the most common for individuals under 50 years of age.^2^ HIV infection may lead to acquired immunodeficiency syndrome (AIDS), a chronic condition that presents not only serious damage to the immune system but also an increased risk of neurocognitive impairment, as the virus is present on the cerebrospinal fluid and spreads on cells of the neurological tissue.^2^ Between 40 to 70% of HIV patients show neurological damage related directly to the infection,^3^ with differing degrees of neurocognitive, motor and neurobehavioral alterations depending mainly on the individual's immunodeficiency levels.^4^ HIV-associated neurocognitive disorders (HAND) may be further classified depending on the severity of neurocognitive impairment and its effects on the patient's daily functioning. Associated-asymptomatic neurocognitive impairment (ANI) is identified when impairment up to 1 standard deviation is found through neuropsychological testing in at least two major cognitive domains, but no loss of functionality is assessed; HIV-1 associated mild cognitive impairment (MND) is assessed when the same neuropsychological standards of ANI are met, but mild impairment to daily functioning, either self-reported or through reliable informants, is identified; and HIV-1 associated dementia (HAD) is diagnosed when neuropsychological performance scores below 2 standard deviations in at least two major cognitive domains and substantial impairment to daily functioning are assessed.^2^ ^,^ ^4^

Further literature on the subject describes that people living with HIV infection initially show cognitive deficits related to attention, memory and recall, and executive functions, most of all working memory.^2^ ^,^ ^5^ Psychomotor alterations, deficits in learning, lower processing speed and speech alterations are expected as the disease progresses.^2^ While less common than in the pre-HAART (highly active antiretroviral therapy) era, motor alterations such as unstable gait, loss of balance, and loss of fine-tuned motor skills, por example, writing, can also be part of the condition.^2^ Though not core traits for diagnosis, other possible associated affective alterations include increased irritation and apathy.^2^

HIV+ patients may also present brain damage caused by opportunistic diseases developed due to their immune-compromised status, such as cerebral toxoplasmosis (CT).^6^ ^–^ ^8^ CT is a possible complication of *Toxoplasma gondii* infection, a common protozoa spread worldwide. About a third of the world's population is possibly infected with *T. gondii*,^8^ with 80% of those as asymptomatic carriers. However, on people living with HIV infection, toxoplasmosis can develop dangerous complications such as seizures, mental confusion and localized neurological deficits.^7^ ^,^ ^8^ Initial manifestations of the infection can show a wide-range of presentations, though a few of the more common include light flu-like symptoms, headaches and fever.^7^ ^,^ ^9^ Further complications can involve seizures, mental confusion, neurological impairment, ataxia, visual abnormalities, cranial nerve palsy and psychomotor or behavioral alterations.^7^ Despite the possible danger it can pose, CT is fully treatable, with the most efficient treatments being the use of sulfadiazine and pyrimethamine, with clyndamycin as an alternative to Sulfadiazine when necessary.^7^ Given the variety of possible symptoms, diagnosis of CT requires confirmation through histological and laboratorial means.^7^ CT patients may show long-lasting cognitive impairment due to brain abscesses, including deficits to the executive functions, lower processing speed and language.^7^ ^,^ ^9^

Another contributing factor to neurocognitive impairment on HIV+ patients worldwide is alcohol abuse.^10^ There is evidence to suggest that consumption of alcohol can impair neurocognitive functioning on both short and long-term, possibly due to the neurotoxicity of alcohol and the negative effects of ethanol on thiamine absorption, a vital substance for neuronal processing.^11^ ^,^ ^12^ In current literature, there is considerable debate on the incidence, epidemiology and diagnosis of alcohol-related dementia (ARD), given that results show heterogeneity of reported symptoms and of related factors such as years of alcohol consumption, pattern of use, comorbidities and sociodemographic characteristics of patients.^12^ According to most authors, ARD usually affects both neurocognitive and neurobehavioral functions, with primary damage to the prefrontal lobe, corpus callosum and the cerebellum, leading in most cases to impairments of executive functioning, working memory, verbal fluency, visuospatial functions and motor control, including slower motor speed and loss of fine level motor skills.^11^ ^,^ ^13^ The most frequent behavioral alterations associated with ARD would be increased social isolation and decreased interest in previous enjoyable activities, similarly to depressed patients.^12^

In discussing ARD, it is important to discuss and distinguish Wernicke-Korsakoff Syndrome (WKS) and Marchiafava-Bignami Disease (MBD), conditions also commonly found on abusive alcohol users. WKS starts as Wernicke's Encephalopathy (WE), characterized by abnormal oculomotor movements, altered mental state (confusion) and dysfunction of the cerebellum,^11^ ^,^ ^12^ leading to impairment of executive functions such as planning, working memory and cognitive flexibility, visuospatial and motor skills. WKS may follow months or years after WE episodes, with patients presenting significative memory impairment that includes anterograde amnesia, with enough strong connection between both to lead researchers to consider WKS a single condition.^11^ ^,^ ^12^

On the other hand, MBD is a rare disease characterized by degeneration of the corpus callosum associated with chronic alcohol abuse and deficiency of B complex vitamins.^14^ It is usually diagnosed by neuroimaging, with signs of degeneration shown by a hypodense corpus callosum,^15^ and its clinical presentation is mixed, including convulsions, depression, aggression, memory deficits, apraxia and gait disorders depending on the severity of the disease.^15^ MBD can be acute, sub-acute or chronic, and usually results in coma, severe dementia or death, with a high mortality rate.^15^

Differential diagnosis between ARD and WKS is debatable, as ARD may share similarly impaired domains and neurological damage with WKS, enough to raise a discussion on whether ARD and WKS would be different presentations of the same condition.^11^ ^–^ ^13^ Currently, this question remains unsolved. As ARD may, according to literature, show enough heterogeneity in presentation to not fully fulfil WKS's core criteria, and to englobe other possible symptomatology,^11^ ^,^ ^12^ for the sake of this case report we refer to both as different conditions.

In non-HIV+ samples, ARD leads to possible impairment of executive functioning, episodic memory, verbal fluency, visuospatial functions and loss of fine motor skills.^11^ ^–^ ^13^ Mild memory impairment, especially to retain new information and retrieve that already stored, can also be more prominent in seropositive alcohol users than on those that do not abuse alcohol.^12^ HIV-alcoholic individuals show greater impairment to executive functions, verbal fluency, visuospatiality, episodic memory and motor speed compared to both on-alcoholic HIV+ patients and non-HIV-alcoholic users.^10^ We found no previous literature on neurocognitive/behavioral alterations on patients that shared all three conditions, raising the question of how such comorbidities would manifest together.

There are also precedents in literature that suggest the relation between HAND, HIV+ infection and vascular damage, as well as vascular dementia (VD).^16^ An inherently heterogenic condition, VD is the second most common type of dementia in the general population, and is frequently comorbid with other conditions.^17^ There are studies which suggest that ischemic damage could be a predictor or associated with HAND,^18^ and a recent proposal suggests that the neurovascular unit would be the prime target during HIV spread in brain tissue, which would cause vascular damage alongside the previously stated neurotoxicity.^16^ It is also important to note how risk factors for vascular dementia, as cardiovascular diseases, are more prominent in individuals living with HIV than in non-HIV+ populations, with the HIV+ population having higher probability of stroke compared to age-matched controls.^18^ ^,^ ^19^ The neurocognitive presentations of HAND and certain types of vascular dementia, such as small vessels disease, can also be quite similar to the point of being neuropsychologically indistinctive in early cases,^20^ including impaired attention, impaired memory, slower mental processes, slower motor skills, irritability and personality changes.^4^ ^,^ ^20^

For this case report, we discuss a possible major neurocognitive disorder due to multiple etiologies, nominally HIV+, CT, vascular and ARD. We review, through the lenses of neuropsychological evaluation, how those multiple factors may have contributed to the patient's neurocognitive and neurobehavioral development.

## CASE REPORT

JS is a right-handed, 63-year-old male, married to the current partner for five years. The patient completed up to the 6^th^ year of the Brazilian Fundamental School, which is considered a primary-level education. Worked as a machine operator, retired seven years prior to the evaluation. The patient and his family gave full permission to publish this case report.

Known medical history includes only hypertension, with no prior or current indication of diabetes, cardiopathy, dyslipidemia, thyroid issues or nephrological issues. In 2018, the patient was admitted to a local hospital after three different episodes of convulsions and vomit on the same month. He was subsequently transferred to a reference infectiology hospital due to suspicion of CT and possible undiagnosed HIV infection. On admission, the patient showed fever (39°C), temporal and spatial disorientation, reduced muscle strength, right side paresis, compromised gait, expression and comprehension aphasia, and confusion, not recognizing his children or following simple commands. Positive diagnosis of both HIV and CT were subsequently confirmed through laboratorial and neuroimaging exams.

Neuroimaging was conducted through magnetic resonance, before and after administration of gadolinium contrast. The initial post-discharge exam shows previously unknown, extensive bilateral damage to the parietal, temporal and occipital areas, both cortical and subcortical, as well as smaller bilateral lesions on the basal ganglia cortical-subcortical transitions and on the parasagittal region of the left cerebellum hemisphere. All aforementioned lesions show evidence of slight periventricular vasogenic edema and small deposits of calcium and blood degradation byproducts, without highlights on post-contrast application nor significant expansive effects. Other physiological alterations include a smaller, localized lesion on the left side of the bulbus, non-hypertensive dilation of the ventricular supratentorial system and compensatory enlargement of brain fissures, cortical sulci and basal cisterns. The diagnostic impression by the imaging professional was that most of the lesions and calcium/blood degradation deposits could be explained by the recent CT infection.

The most recent exam, from January 2020, confirms most of those original findings, but points further details and raises different explanations for their possible origins. This new exam identifies most of the extensive damage to the parietal, temporal and occipital areas as localized to the left hemisphere, with smaller, bilateral lesions to the cortical-subcortical connections on the frontal region and basal ganglia. Additionally, the white matter shows diffuse hypersignal focus on repetition time (TR) sequences, suggestive of microangiopathy, that were not identified in previous exams. The diagnostic impression of this new exam suggests that the damage to parietal, temporal and occipital areas on the left hemisphere is likely ischemic in origin, with the bilateral damage basal ganglia and the cortical-subcortical transitions being possible consequences of the CT infection.

JS stayed at the hospital for 42 days for treatment of the *T. gondii* infection, with the use of clindamycin and corticotherapy for 34 days and prednisone for 7 days, due to allergic reaction to sulfadiazine. On hospital discharge, JS presented negative exams to syphilis, hepatitis B and C, Venereal Disease Research Laboratory (VDRL), leveduriform structures, and absence of alcohol acid resistant bacilli (BAAR) per Ziehl Neelsen's Bacilloscopy. GeneXpert Test and Quick Molecular Test for tuberculosis were both undetectable. Patient's viral charge was of 650.033 copies, with CD4 of 59. On cognitive evaluation during discharge, JS was able to recognize family members, articulate meaningful short phrases, walk with assistance, and had reduced paresis, but remained temporal and spatially disoriented.

After returning home, the patient initiated HAART through the use of Tenovir Desoproxil Fumarate+Lamivudine and Raltegravir, as well as neurological follow-ups to treat the aforementioned motor and language alterations. Besides HAART medication, the patient is also is constant use of Donaren 50 mg (antidepressive) and Cinetol 100 mg (antiparkisonism), both prescribed by neurology. Prior to evaluation, the patient made use of folic acid for a year, and was no longer considered B9 vitamin deficient by the time he arrived at our service.

In 2019, JS was referred to our neuropsychology service, Service of Neuropsychology of Aging (Serviço de Neuropsicologia do Envelhecimento -SENE/UFRN), for further evaluation of cognitive domains and behavior. Because of the patient's language and memory impairment, the anamnesis was conducted with the help of his wife, who had no knowledge on his family history. On follow-up sessions, JS's communication improved, allowing us to confirm most of her testimonies. Unfortunately, no other sources on his personal history were available.

### Neurocognitive alterations

The main reports of cognitive deficits on JS's case were of temporal and spatial disorientation, short-term and anterograde memory loss, expression aphasia and inability to write. During evaluation, the patient showed consistent difficulty to remember the date, year and location, being only able to identify he was at a hospital. He recognized the technicians and the location on following appointments, though not by name.

JS could appropriately understand and answer to requests, but showed difficulty to express his own thoughts and feelings. Especially during the anamnesis, the patient communicated with little fluency, using short, simple sentences, and needing his wife's help to express more complex concepts. This is a similar, if considerably improved, aphasic presentation to what was identified by the medical professionals on his hospital admission. Further into the evaluation, the patient became more talkative and formed longer sentences, but still showed little fluency and fragmented discourse, as well as impairment of semantic memory. Linguistically, the patient's vocabulary and syntax did not differ from the expected for his age and scholarly level. Considering the evidence of extensive cortical and subcortical damage on localized on the left hemisphere, a pattern previously associated with aphasia in literature,^21^ we suggest the possibility that JS's aphasia presentation is related to the ischemic damage pointed out by his neuroimage exams.

In regards to memory impairment, JS had consistent difficulty in recall when asked about past memories, explaining that “he did not remember” most of what was questioned. He was still capable of recognizing family and friends, with difficulty naming them. Unprompted, he could recall past episodes without chronological order. Most of the childhood and early adulthood memories were intact, with impaired recall on memories of later years.

Beyond the aforementioned issues, JS showed severe visuospatial impairment throughout the whole evaluation, consistently struggling with visuospatial tests and simple tasks like estimating the distance to several objects. The patient also showed considerable impairment to attention and executive functions, most of all working memory and cognitive flexibility.

### Neurobehavioral alterations

Throughout the evaluation period, JS avoided direct eye contact, verbally denied answers or requests, gave up on tasks easily and showed anxious signs, such as mouth tremors while performing tasks. All the behavioral traits mentioned occur in depressive patients,^22^ as well as on patients with moderate to severe dementia.^23^ Despite JS's final score on both instruments being mixed, we cannot assess that those behavioral traits were due to a depressive disorder. It is important to note that both instruments were applied as part of our Service's standard neuropsychological battery for newcomer patients, and that the patient was referred to psychiatric evaluation at his last session.

**Figure 1:**
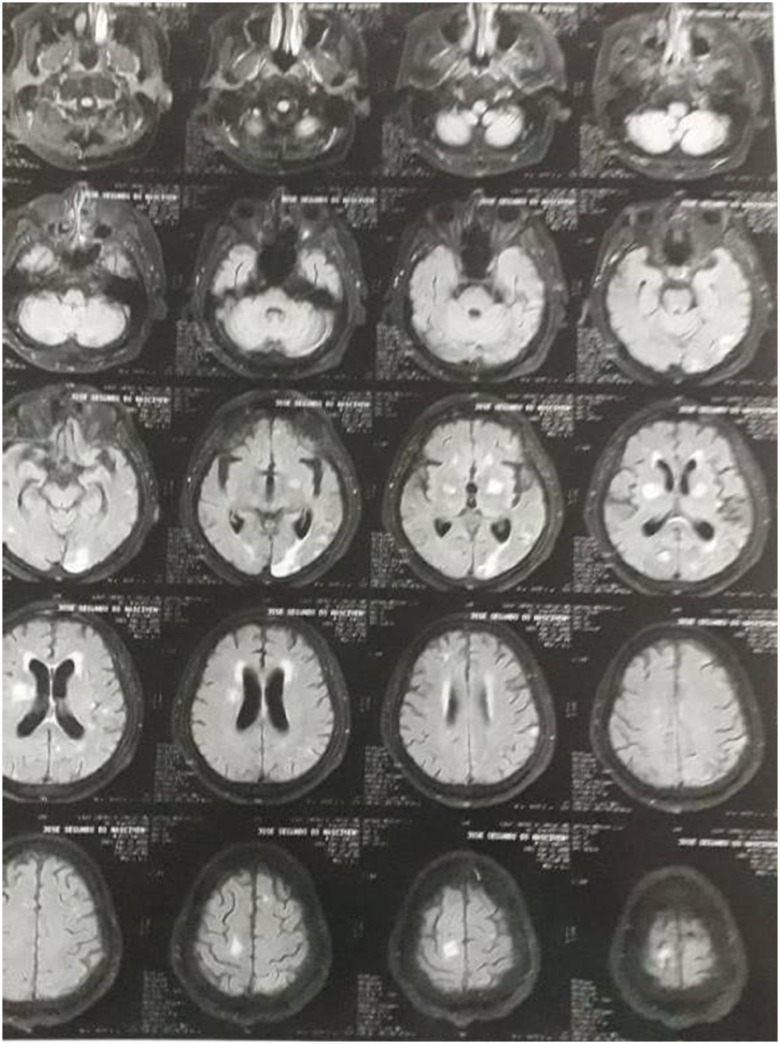
Patient's original neuroimaging scan shows extensive bilateral damage to parietal, temporal and occipital areas, cortical and subcortical, smaller bilateral lesions on the basal ganglia cortical-subcortical transitions and on the parasagittal region of the left cerebellum hemisphere. All lesions show evidence of slight periventricular vasogenic edema and deposits of calcium and blood degradation byproducts, without highlights post-contrast application nor significant expansive effects. Other physiological alterations include a localized lesion on the left side of the bulbus, non-hypertensive dilation of the ventricular supratentorial system and compensatory enlargement of brain fissures, cortical sulci and basal cisterns. The diagnostic impression by the imaging professional is that most of the lesions and calcium/blood degradation deposits could be explained by the recent (at the time) cerebral toxoplasmosis infection.

**Figure 2:**
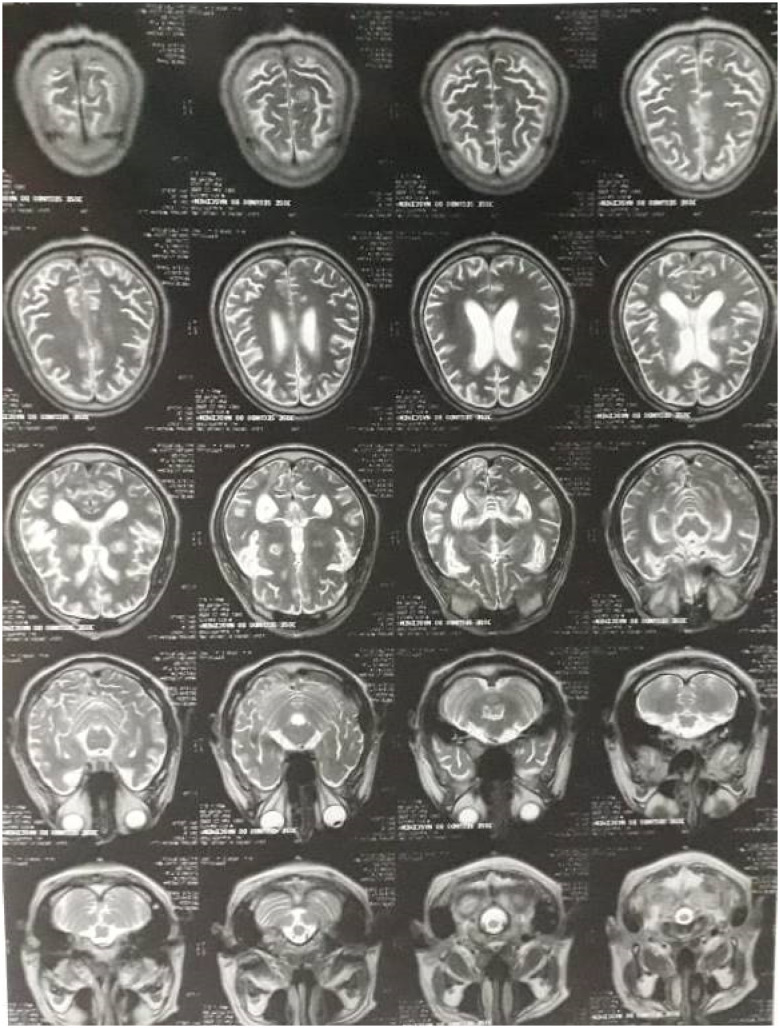
JS's most recent neuroimaging scan, from January 2020, confirms most of the above findings but diverges in key points. The new exam localizes most of the extensive damage to the parietal, temporal and occipital areas on the left hemisphere, with smaller bilateral lesions to the cortical-subcortical connections mostly localized to the frontal region and basal ganglia. Additionally, there are hypersignal focus on TR sequences diffused throughout the white matter, a suggestion of microangiopathy not identified in previous exams. The diagnostic impression suggests that the damage to parietal, temporal and occipital areas on the left hemisphere is likely ischemic in origin and pre-infectious, with the damage to the basal ganglia and the cortical-subcortical transitions being possible consequences of the cerebral toxoplasmosis infection.

**Figure 3:**
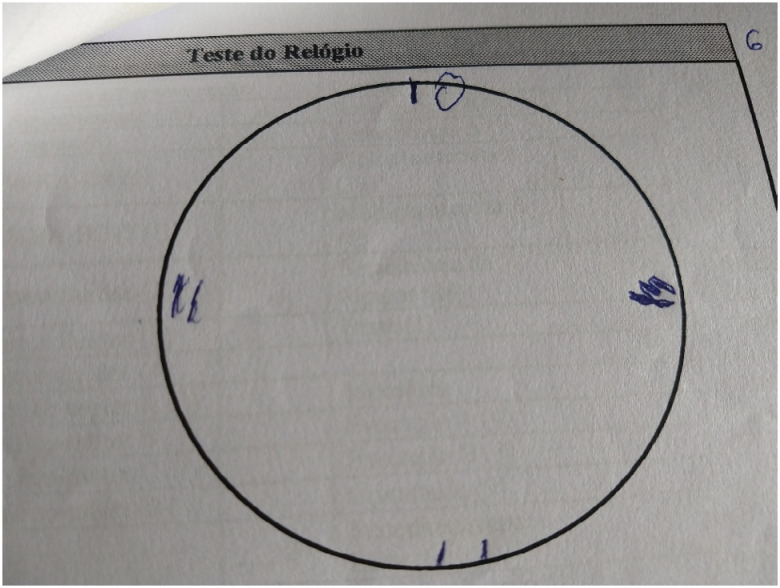
JS's clock drawing test, showing severe visuospatial and executive functioning impairment.

Further medical history revealed that JS made daily and heavy use of alcohol for several years, with neither him nor his wife precising the age at which it started. This continued up to the first hospital admission, with the patient consuming alcohol between the three convulsive episodes. At the time of the evaluation, the patient was abstemious for one year (Table 1 and Figures 1, 2 and 3).

**Table 1:** Neuropsychological instruments and patient scores.

Instruments applied (tests and scales)		Scores*
Montreal Cognitive Assessment (MoCA)		4/30
Clock Drawing Test		0/5
*Digit Span*		
*Direct Order*		
	Correct sequences	02/16
	Total score (span x correct seq.)	004/144
*Inverse Order*		
	Correct sequences	00/14
	Total score (span x correct seq.)	000/112
Frontal Assessment Battery		5/18
Five Digit Test		
	Reading	166”
	Counting	161”
	Election	168”
	Alternance	287”
	Inhibition	002”
	Flexibility	121”
Phonemic Verbal Fluency Test		01
Stroop Test		
	*First Card*	103s
	*Second Card*	71s
	*Third Card*	146s
Semantics Verbal Fluency Test		
	Animals	03
Boston Naming Test		07/15
Construction Subscale (DRS – 2)		0/6
Stick Construction Test		4/12
9 Hole Peg Test		
	*Non-Dominant Hand*	65s
	*Dominant Hand*	79s
Corsi Cubes Test		
*Direct Order*		
	Correct sequences	00/16
	Total score (span x correct seq.)	00/144
*Inverse Order*		
	Correct sequences	0/14
	Total score (span x correct seq.)	0/112
Beck Depression Inventory		24/30
Geriatric Depression Scale		04/15
Pfeffer's Functional Activities Questionnaire		17/30
Berg Balance Scale		37/56
Functional Independence Measure Scale		
	*Patient's self-evaluation*	88/126
	*Caretaker's evaluation*	117/126

* Scores of all cognitive tests indicated neurocognitive impairment according to published normative data for standardized tests.

## DISCUSSION

After CT, JS presented an acute decline from previous levels of functioning, being unable to perform daily activities. We identified major deficits to visuospatial abilities, executive functions (working memory and cognitive flexibility) and attention, episodic and semantic memory, and lower brain processing speed. Non-cognitive symptomatology includes parkinsonism, bradykinesia, loss of fine motor skills and expression aphasia, which is congruent not only with CT-related and vascular brain damage but also with HAD criteria and ARD.

According to Eggers et al.^2^ and Clifford and Ances,^5^ some of the initial cognitive impairments in a HAD diagnosis would be impairment to executive functions (most of all working memory), attention, and memory and recall, including learning, all of which are present in JS's case. The patient also shows subcortical brain damage in a pattern that is associated with certain HAND presentations,^24^ as well as acute loss of functioning which is fundamental for this diagnosis.^2^ However, JS also presents significant symptoms associated with CT infection, such as impairment of verbal episodic memory and lower processing speed, and others associated with ARD, such as impaired verbal fluency, deficits to episodic memory, impaired cognitive flexibility and loss of fine motor skills.

Though the anterograde amnesia would be a strong point towards WKS, we assessed that the patient did not fulfil the criteria for this condition due to lack of evidence of any previous episodes of WE, as well as no reports of any sort of oculomotor abnormalities or altered mental states outside of confusion noticed on the CT infection admission, and that remised with CT treatment. We also assessed that the patient did not show the appropriate neuroimaging results to fit a MBD diagnosis. Per our reasoning, the patient's impairments were better understood through the lenses of ARD than WKS or MBD.

Considering the difficulty in assessing an accurate timeline of the patient's medical history, including more precise dates for the beginning of the CT and HIV infections, and the years of alcohol abuse, as well as taking into account the extensive vascular-related neuroimage evidence, HAD-related history, ARD neurotoxicity damage and further CT damage to the basal ganglia, we reasoned then that the diagnosis of dementia due to multiple etiologies (DME) would better contemplate the patient's clinical condition than pointing only one of those etiologies as the main contributor. We consider that there is a strong indication that all the aforementioned pathologies contributed to the acute loss of functionality that JS experienced after the hospital admission, with earlier symptomatology likely going unnoticed by both the patient and his wife, or attributed by both to a healthy aging process.

It is important to explore in literature the frequent, but underreported, condition of DME. While comorbidity of different pathologies is not statistically unusual in dementia cases,^1^ such comorbidities are usually confirmed post-mortem, when a more thorough examination is possible. Even for conditions that could be diagnosed in life, such as mixed dementia (comorbid between Alzheimer's and Vascular Disease),^25^ it is more common for professionals to focus on a single major probable or possible factor, which can lead to an incomplete understanding of the symptomatology and cognitive impairment of the patient, as well as poor comprehension for neuropsychologists and other health professionals on the possible presentations of DME. Considering the importance of differential diagnosis for the prognosis and quality of life of patients,^26^ identifying the influence of multiple etiologies in cognitive impairment is an integral step for proper management of treatment possibilities, especially in cases of multiple domain dysfunction and overlapping conditions such as that of JS.

This case report illustrates a DME condition in which several factors contributed to an acute cognitive decline with serious consequences to the patient's daily functioning and quality of life. We consider this information helpful for recognition of cognitive impairment in DME patients, for the management of similarly comorbid cases in third world countries, and for future differential diagnosis of neurocognitive impairment for people living with HIV infection.
